# Extreme values of derivatives of the Riemann zeta function

**DOI:** 10.1112/mtk.12130

**Published:** 2022-04-16

**Authors:** Daodao Yang

**Affiliations:** ^1^ Institute of Analysis and Number Theory Graz University of Technology Graz Austria

## Abstract

It is proved that if *T* is sufficiently large, then uniformly for all positive integers $\ell ⩽\left(\log T\right)/\left(\log_{2}T\right)$, we have

$$\begin{array}{ccc} & & \;\max_{T⩽t⩽2T}\left|\zeta^{\left(\ell \right)}\left(1+it\right)\right|⩾e^{\gamma }\cdot \ell^{\ell }\cdot {\left(\ell +1\right)}^{-(\ell +1)}\\ & & \;\;\cdot {\left(\log_{2}T-\log_{3}T+O\left(1\right)\right)}^{\ell +1}\;, \end{array}$$

where γ is the Euler constant. We also establish lower bounds for maximum of $|\zeta^{\left(\ell \right)}\left(\sigma +it\right)|$ when ℓ∈N and σ∈[1/2,1) are fixed.

## 1 INTRODUCTION

This paper establishes the following new results for extreme values of derivatives of the Riemann zeta function (in this paper, we use the short‐hand notations, $\;\log_{2}T:=\log\log T$, and $\log_{3}T:=\log\log\log T$).

Theorem 1 If *T* is sufficiently large, then uniformly for all positive integers $\ell ⩽\left(\log T\right)/\left(\log_{2}T\right)$, we have

$$\max_{T⩽t⩽2T}\left|\zeta^{\left(\ell \right)}\left(1+it\right)\right|⩾e^{\gamma }\cdot \ell^{\ell }\cdot {\left(\ell +1\right)}^{-(\ell +1)}\cdot {\left(\log_{2}T-\log_{3}T+O\left(1\right)\right)}^{\ell +1}\;.$$

Remark 1 In our Theorem 1, ℓ does not have to be fixed. In particular, if $\ell =[\left(\log T\right)/\left(\log_{2}T\right)]$, then for sufficiently large *T*, we have

$$\max_{T⩽t⩽2T}\left|\zeta^{\left(\ell \right)}\left(1+it\right)\right|\gg \exp\{\frac{\log T}{\log_{2}T}\left(\log_{3}T\right)-4\;\frac{\log T}{{\left(\log_{2}T\right)}^{2}}\left(\log_{3}T\right)\}.$$

This value is even larger than the conditional upper bound of extreme value of the Riemann zeta function on the $\frac{1}{2}$‐line in the same interval [T,2T]. Recall that Littlewood [20] proved that the Riemann hypothesis *(*RH*)* implies the existence of a constant *C* such that for large *T* we have $\;\;\max_{T⩽t⩽2T}\left|\zeta \left(\frac{1}{2}+it\right)\right|$ ≪exp{C(logT) $\cdot {\left(\log_{2}T\right)}^{-1}\}$. Chandee and Soundararajan [10] proved that on RH, one can take any constant C>(log2)/2.

Theorem 2 Let ℓ∈N and β∈[0,1) be fixed.

(A) Let *c* be a positive number less than $\sqrt{2(1-\beta )}$. If *T* is sufficiently large, then
$$\max_{T^{\beta }⩽t⩽T}\left|\zeta^{\left(\ell \right)}\left(\frac{1}{2}+it\right)\right|⩾\exp\{c\sqrt{\frac{\log T\;\log_{3}T}{\log_{2}T}}\}.$$
(B) Let $\sigma \in (\frac{1}{2},1)$ be given and κ be a positive number less than 1−β. Then for sufficiently large *T*, we have
$$\max_{T^{\beta }⩽t⩽T}\left|\zeta^{\left(\ell \right)}\left(\sigma +it\right)\right|⩾\exp\{\frac{\tilde{c}\cdot \kappa^{1-\sigma }}{\left(1-\sigma \right)}\cdot \frac{{\left(\log T\right)}^{1-\sigma }}{{\left(\log_{2}T\right)}^{\sigma }}\}\;,$$
  where $\tilde{c}$ is an absolute positive constant.

Remark 2 By Soundararajan's original resonance method [22], we can also establish lower bounds for the maximum of derivatives of the zeta function on the $\frac{1}{2}$‐line on the shorter interval [T/2,T]. In this case we obtain $\max_{T/2⩽t⩽T}\left|\zeta^{\left(\ell \right)}\left(\frac{1}{2}+it\right)\right|⩾\exp\left\{\left(1+o\left(1\right)\right)\sqrt{\log T/\log_{2}T}\right\}$, losing a $\;\log_{3}T$ factor compared to the above result on the longer interval $\left[T^{\beta },T\right]$.

The research for extreme values of the Riemann zeta function has a long history. In 1910, Bohr and Landau first established the result $\zeta \left(1+it\right)=\Omega \left(\log_{2}t\right)$ (see [23, Theorem 8.5]). In 1924, Littlewood (see [23, Theorem 8.9(A)]) was able to find an explicit constant in the Ω‐result of Bohr and Landau, by proving that ${\bar{\lim}}_{t\to \infty }\left|\zeta \left(1+it\right)\right|/\left(\log_{2}t\right)⩾e^{\gamma }$. Littlewood's result was improved by Levinson [18] in 1972, and by Granville–Soundararajan [14] in 2005. The currently best‐known lower bound is established by Aistleitner–Mahatab–Munsch [3] in 2017, who proved that $\max_{\sqrt{T}⩽t⩽T}\left|\zeta \left(1+it\right)\right|⩾e^{\gamma }\left(\log_{2}T+\log_{3}T-C\right)$, for some constant *C*.

On the other hand, when assuming the RH, Littlewood proved that $\left|\zeta \left(1+it\right)\right|⩽\left(2e^{\gamma }+o\left(1\right)\right)\log_{2}t\;$, for sufficiently large *t* (see [23, Theorem 14.9]). Furthermore, Littlewood conjectured that $\max_{1⩽t⩽T}\left|\zeta \left(1+it\right)\right|∼e^{\gamma }\log_{2}T$. In [14], Granville–Soundararajan made the stronger conjecture:  $\max_{T⩽t⩽2T}\left|\zeta \left(1+it\right)\right|=e^{\gamma }\left(\log_{2}T+\log_{3}T+C_{1}\right)+o\left(1\right)$, for some constant *C*_1_ which can be effectively computed.

Compared to the research on extreme values of the Riemann zeta function, much less is known about the extreme values of its derivatives. In [17], Kalmynin obtained Ω‐results for the Riemann zeta function and its derivatives in some regions inside the critical strip near the line ℜ(s) = 1. He also mentioned that his methods do not provide any non‐trivial results about the domains of the form σ⩾σ(t) with σ(t)=1−o(logloglogt/loglogt). Note that Kalmynin did not obtain Ω‐results for $|\zeta^{\left(\ell \right)}\left(\sigma +it\right)|$ when ℓ∈N and σ∈[1/2,1) are given.

It is still uncertain whether the methods of [3, 14, 18, 23] are able to establish the result in our Theorem 1, since those methods basically rely on the fact that the *k*‐divisors function $d_{k}\left(n\right)$ is multiplicative and/or the fact that the Riemann zeta function has an Euler product: $\zeta \left(s\right)=\prod_{p}{\left(1-p^{-s}\right)}^{-1}$, ℜ(s)>1. Note that the function $f\left(n\right):={\left(\log n\right)}^{\ell }$ is not multiplicative and the derivative $\zeta^{\left(\ell \right)}\left(s\right)$ does not have an Euler product.

We also emphasize that the key points in Theorem 1 are the range $\ell ⩽\left(\log T\right)/\left(\log_{2}T\right)$ and the constant in front of ${\left(\log_{2}T\right)}^{\ell +1}$. In fact, one can use the method of Bohr–Landau to prove a much weaker result, that is, $\zeta^{\left(\ell \right)}\left(1+it\right)=\Omega \left({\left(\log_{2}t\right)}^{\ell +1}\right)$ when ℓ∈N is fixed. See Section 7 for such a short proof.

We will use Soundararajan's original resonance method [22] to prove Theorem 1. The new ingredient for the proof is the following Proposition 1.

Proposition 1 If *T* is sufficiently large, then uniformly for all positive numbers ℓ, we have

$$\underset{r}{\sup}\left|\;\sum_{mk=n⩽\sqrt{T}}\frac{r\left(m\right)\bar{r(n)}}{k}{\left(\log k\right)}^{\ell }\;\right|/\left(\;\sum_{n⩽\sqrt{T}}{\left|r\left(n\right)\right|}^{2}\;\right)⩾\frac{e^{\gamma }}{\ell }\cdot {\left(\;\frac{\ell }{\ell +1}\;\right)}^{\ell +1}\cdot {\left(\log_{2}T-\log_{3}T+O\left(1\right)\right)}^{\ell +1},$$

where the supremum is taken over all functions r:N→C satisfying that the denominator is not equal to zero, when the parameter *T* is given.

The following Proposition 2 will not be used to prove our theorems. However, it is closely related to Proposition 1 and can be viewed as a “log‐type” greatest common divisor (GCD) sum, so we list it here for independent interest.

Proposition 2 Let ℓ∈(0,∞) and let $c_{\ell }$ be a positive number less than $\;\;6e^{2\gamma }\pi^{-2}\ell^{2\ell }{\left(2\ell +1\right)}^{-(2\ell +1)}\;$.  For sufficiently large *N*, we have

$$\underset{|M|=N}{\sup}\sum_{m,n\in M}\frac{\left(m,n\right)}{\left[m,n\right]}\log^{\ell }\left(\frac{m}{\left(m,n\right)}\right)\log^{\ell }\left(\frac{n}{\left(m,n\right)}\right)⩾c_{\ell }\cdot N\cdot {\left(\log_{2}N\right)}^{2+2\ell }\;,$$

where the supremum is taken over all subsets M⊂N with size *N*.

Remark 3 Actually we can also use Proposition 2 and Hilberdink's version of the resonance method [15] to prove a similar result to the one in Theorem 1. But the constant in front of $\;\;{\left(\log_{2}T\right)}^{\ell +1}$ will be much worse.

Soundararajan introduced his resonance method in [22] and proved that

$$\max_{T⩽t⩽2T}\left|\zeta \left(\frac{1}{2}+it\right)\right|⩾\exp\left(\left(1+o\left(1\right)\right)\sqrt{\frac{\log T}{\log\log T}}\right),$$

which improved earlier results of Montgomery and Balasubramanian–Ramachandra. Montgomery [21] proved it under RH and with the constant 1/20 instead of 1+o(1) in Soundararajan's result. Balasubramanian–Ramachandra [4] proved the result unconditionally but also with a smaller constant compared to Soundararajan's result.

By constructing large GCD sums, Aistleitner [1] used a modified version of Soundararajan's resonance method to establish lower bounds for maximum of |ζ(σ+it)| when σ∈(1/2,1) is fixed. His results improved early results of Voronin [24] and Hilberdink [15] via resonance methods. He proved that

$$\max_{0⩽t⩽T}\left|\zeta \left(\sigma +it\right)\right|⩾\exp\left(\frac{c_{\sigma }{\left(\log T\right)}^{1-\sigma }}{{\left(\log\log T\right)}^{\sigma }}\right),$$

for large *T*, and one can take $c_{\sigma }=0.18{\left(2\sigma -1\right)}^{1-\sigma }$. The same result had been proved by Montgomery in [21] with a smaller value for $c_{\sigma }$. In [9], Bondarenko and Seip improved the value $c_{\sigma }$ in Aistleitner's result.

By constructing large GCD sums, using a convolution formula for ζ in the resonance method, Bondarenko and Seip [7, 8] proved the following surprising result:

$$\max_{1⩽t⩽T}\left|\zeta \left(\frac{1}{2}+it\right)\right|⩾\exp\left(\left(1+o\left(1\right)\right)\sqrt{\frac{\log T\log_{3}T}{\log_{2}T}}\right).$$

After optimizing the GCD sums, de la Bretèche and Tenenbaum [11] improved the factor from (1+o(1)) to $\left(\sqrt{2}+o\left(1\right)\right)$ in the above result.

Following the work of Bondarenko–Seip and de la Bretèche–Tenenbaum, we use their modified versions of resonance methods to prove Theorem 2. The new ingredient is our convolution formula for $1+2^{-s}+{\left(-1\right)}^{\ell }\zeta^{\left(\ell \right)}\left(s\right)$. Throughout the paper, define the function $F_{\ell }\left(s\right)$ as follows:

(1) $$F_{\ell }\left(s\right):=1+\frac{1}{2^{s}}+{\left(-1\right)}^{\ell }\zeta^{\left(\ell \right)}\left(s\right).$$

Throughout the paper, also define the sequence ${\left\{a_{\ell }\left(n\right)\right\}}_{n=1}^{\infty }$ as $a_{\ell }\left(1\right)=1,a_{\ell }\left(2\right)=1+{\left(\log2\right)}^{\ell }$, and $a_{\ell }\left(n\right)={\left(\log n\right)}^{\ell }$ for n⩾3. Then we have the following identity and the Dirichlet series converge absolutely:

(2) $$F_{\ell }\left(s\right)=\sum_{n=1}^{\infty }\frac{a_{\ell }\left(n\right)}{n^{s}},\;\;ℜ\left(s\right)>1.$$

The reason why we add the part $1+2^{-s}$ is that we want to make $a_{\ell }\left(n\right)⩾1$ for all n⩾1. Since when σ∈[1/2,1), the factor ${\left(\log n\right)}^{\ell }$ has very small influence on the log‐type GCD sums compared to the case σ=1, we will simply use the fact that $a_{\ell }\left(n\right)⩾1$ and then come to the situation of optimizing GCD sums.

Let σ∈(0,1] be given and let M⊂N be a finite set. The GCDs sums $S_{\sigma }\left(M\right)$ of M are defined as follows:

$$\begin{array}{c} S_{\sigma }\left(M\right):=\sum_{m,n\in M}\frac{{\left(m,n\right)}^{\sigma }}{{\left[m,n\right]}^{\sigma }},\; \end{array}$$

where (m,n) denotes the GCD of *m* and *n* and [m,n] denotes the least common multiple of *m* and *n*.

The case σ=1 was studied by Gál [13], who proved that

(3) $$\begin{array}{c} {\left(\log_{2}N\right)}^{2}\ll \underset{|M|=N}{\sup}\frac{S_{1}\left(M\right)}{\left|M\right|}\ll {\left(\log_{2}N\right)}^{2}. \end{array}$$

The asymptotically sharp constant in (3) is $6e^{2\gamma }\pi^{-2}$. This fact was proved by Lewko and Radziwiłł   in [19].

Bondarenko and Seip [6, 7] proved the following result for GCD sums when $\sigma =\frac{1}{2}:$

$$\begin{array}{c} exp\{\left(1+o(1)\right)\sqrt{\frac{\log N\;\log_{3}N}{\log_{2}N}}\}\ll \underset{|M|=N}{\sup}\frac{S_{\frac{1}{2}}\left(M\right)}{\left|M\right|}\ll exp\{\left(7+o(1)\right)\sqrt{\frac{\log N\;\log_{3}N}{\log_{2}N}}\}. \end{array}$$

Later, based on constructions of [6, 7], de la Bretèche and Tenenbaum [11] optimized the result of Bondarenko–Seip and obtained the following:

(4) $$\begin{array}{c} \underset{|M|=N}{\sup}\frac{S_{\frac{1}{2}}\left(M\right)}{\left|M\right|}=exp\{\left(2\sqrt{2}+o\left(1\right)\right)\sqrt{\frac{\log N\;\log_{3}N}{\log_{2}N}}\}. \end{array}$$

Aistleitner, Berkes, and Seip [2] proved the following essentially optimal result for GCD sums when $\sigma \in (\frac{1}{2},1)$, where $c_{\sigma }$ and $C_{\sigma }$ are positive constants only depending on σ:

(5) $$\begin{array}{c} exp\{c_{\sigma }\cdot \frac{{\left(\log N\right)}^{1-\sigma }}{{\left(\log_{2}N\right)}^{\sigma }}\}\ll \underset{|M|=N}{\sup}\frac{S_{\sigma }\left(M\right)}{\left|M\right|}\ll exp\{C_{\sigma }\cdot \frac{{\left(\log N\right)}^{1-\sigma }}{{\left(\log_{2}N\right)}^{\sigma }}\}. \end{array}$$

Moreover, in [2, p. 1526], they also gave an example (following ideas of [13]) for the lower bound when $\sigma \in (\frac{1}{2},1)$. Let $N=2^{r}$ and let M be the set of all square‐free integers composed of the first *r* primes. Then

(6) $$\begin{array}{c} \frac{S_{\sigma }\left(M\right)}{\left|M\right|}\gg exp\{\frac{\tilde{c}}{1-\sigma }\cdot \frac{{\left(\log N\right)}^{1-\sigma }}{{\left(\log_{2}N\right)}^{\sigma }}\} \end{array}$$

for some positive constant $\tilde{c}$. For simplicity, in our proof we will use this construction. For more constructions, see Bondarenko–Seip [9, pp. 131–136] and Z. Dong‐B.Wei [12, Theorem 1.2].

## 2 LEMMAS FOR THE RIEMANN ZETA FUNCTION

Lemma 1 Let $\sigma_{0}\in \left(0,1\right)$ be fixed. If *T* is sufficiently large, then uniformly for ε>0, t∈[T,2T], $\sigma \in [\sigma_{0}+\varepsilon ,\;\infty )$ and all positive integers ℓ, we have

(7) $${\left(-1\right)}^{\ell }\zeta^{\left(\ell \right)}\left(\sigma +it\right)=\sum_{n⩽T}\frac{{\left(\log n\right)}^{\ell }}{n^{\sigma +it}}+O\left(\frac{\ell !}{\varepsilon^{\ell }}\cdot T^{-\sigma +\varepsilon }\right)\;,$$

where the implied constant in big O(·) only depends on σ_0_.

It follows from Hardy–Littlewood's classical approximation formula (see [23, Theorem 4.11]) for ζ(s) and Cauchy's integral formula for derivatives.□

Lemma 2 Let ℓ∈N and ε∈(0,1) be fixed. Then uniformly for all |t|⩾1 and σ∈[−ε,1+ε],

(8) $$\zeta^{\left(\ell \right)}\left(\sigma +it\right)\ll {\left|t\right|}^{\frac{1-\sigma +3\varepsilon }{2}},$$

where the implied constant depends on ℓ and ε only.

It follows from classical convex estimates for ζ(s) and Cauchy's integral formula.□

In the following, we will derive a “double version” convolution formula, similar to Lemma of 5.3 of de la Bretèche and Tenenbaum [11]. The proof is same as the proof of “single version” convolution formulas in Lemma 1 of Bondarenko and Seip [8].

Define the Fourier transform $\hat{K}$ of *K* as

$$\hat{K}\left(\xi \right):=\int_{-\infty }^{\infty }K\left(x\right)e^{-ix\xi }dx.$$

Lemma 3 Let ℓ∈N and σ∈[0,1) be fixed. Write z=x+iy. Assume that K(z) is a holomorphic function in the strip y=ℑz∈[σ−2,0], satisfying the growth condition

(9) $$\max_{\sigma -2⩽y⩽0}|K\left(z\right)|=O\left(\frac{1}{x^{2}+1}\right).$$

If t∈R∖{0}, then

(10) $$\begin{array}{cc} \int_{-\infty }^{\infty }F_{\ell }\left(\sigma +it+iy\right)F_{\ell }\left(\sigma -it+iy\right)K\left(y\right)dy= & \sum_{m,n⩾1}\frac{\hat{K}\left(\log nm\right)}{n^{\sigma +it}\cdot m^{\sigma -it}}a_{\ell }\left(n\right)a_{\ell }\left(m\right)\\ & -2\pi (\Delta^{+}+\Delta^{-})\ell !, \end{array}$$

where

(11) $$\begin{array}{c} \Delta^{+}=\sum_{\begin{array}{c} m+n=\ell \\ m,n⩾0 \end{array}}\frac{1}{m!n!}{\left(\frac{d}{dz}\right)}^{m}F_{\ell }\left(z+it\right){{|}_{z=1+it}\cdot {\left(\frac{d}{dz}\right)}^{n}K\left(i\sigma -iz\right)|}_{z=1+it} \end{array}$$

and

(12) $$\Delta^{-}=\sum_{\begin{array}{c} m+n=\ell \\ m,n⩾0 \end{array}}\frac{1}{m!n!}{\left(\frac{d}{dz}\right)}^{m}F_{\ell }\left(z-it\right){{|}_{z=1-it}\cdot {\left(\frac{d}{dz}\right)}^{n}K\left(i\sigma -iz\right)|}_{z=1-it}.$$

Define $h\left(z\right):=F_{\ell }\left(z+it\right)F_{\ell }\left(z-it\right)K\left(i\sigma -iz\right)$. h(z) is a meromorphic function in the vertical strip σ⩽ℜ(z)⩽2, with two poles, namely, at z=1+it and z=1−it. Let *Y* be large and consider straight line integrals for h(z). Set $J_{1}=\int_{\sigma -iY}^{2-iY}h\left(z\right)dz,\;J_{2}=\int_{2-iY}^{2+iY}h\left(z\right)dz,\;J_{3}=\int_{2+iY}^{\sigma +iY}h\left(z\right)dz,\;J_{4}=\int_{\sigma +iY}^{\sigma -iY}h\left(z\right)dz$.

Note that $F_{\ell }\left(s\right)=\ell !/{\left(s-1\right)}^{\ell +1}+E\left(s\right)$, where E(s) is an entire function. The residue theorem gives that

(13) $$J_{1}+J_{2}+J_{3}+J_{4}=2\pi i\left(\Delta^{+}+\Delta^{-}\right)\ell !.$$

By (2) and (9) and applying Cauchy's theorem term by term,

$$\lim_{Y\to \infty }J_{2}=i\sum_{n=1}^{\infty }\sum_{m=1}^{\infty }a_{\ell }\left(n\right)a_{\ell }\left(m\right)\frac{\hat{K}\left(\log nm\right)}{n^{\sigma +it}\cdot m^{\sigma -it}}.$$

Clearly,

$$\lim_{Y\to \infty }\left(-J_{4}\right)=i\int_{-\infty }^{\infty }F_{\ell }\left(\sigma +it+iy\right)F_{\ell }\left(\sigma -it+iy\right)K\left(y\right)dy.$$

By the trivial estimate $F_{\ell }\left(s\right)\ll 1+\left|\zeta^{\left(\ell \right)}\left(s\right)\right|$, estimates for $\zeta^{\left(\ell \right)}\left(s\right)$ (Lemma 2) and (9), we obtain

$$J_{1}\ll_{\varepsilon }\frac{1}{Y^{2}}\left(1+\int_{\sigma }^{1+\varepsilon }\left(1+Y^{1-x+3\varepsilon }\right)dx\right)\ll_{\varepsilon }\frac{1}{Y^{2}}\left(1+\frac{Y^{1+3\varepsilon -\sigma }-Y^{2\varepsilon }}{\log Y}\right).$$

Take ε=1/6, then $J_{1}\ll 1/\left(\sqrt{Y}\log Y\right)\to 0$, as Y→∞. Similarly for *J*_3_.□

The following results are due to Hadamard, Landau, and Schnee (also see [16]).

Lemma 4 (Hadamard, Landau, and Schnee) Let μ,ν∈N and $\alpha_{1},\alpha_{2}\in \left(-\frac{1}{2},\infty \right)$ be fixed. Suppose $\alpha_{1}+\alpha_{2}>1$, then

$$\int_{1}^{T}\zeta^{\left(\mu \right)}\left(\alpha_{1}\;+\;it\right)\;\zeta^{\left(\nu \right)}\left(\alpha_{2}\;-\;it\right)dt∼\zeta^{\left(\mu +\nu \right)}\left(\alpha_{1}+\alpha_{2}\right)T,\;\;as\;\;T\to \infty .$$

Remark 4 In this paper, if it is not stated, the limit notations “∼” and “*o*()” are under the condition when the corresponding variable tends to infinity. Namely, as T→∞, t→∞, or N→∞.

In particular, when ℓ∈N and $\sigma \in (\frac{1}{2},1)$ are fixed, one has

(14) $$\int_{0}^{T}|\zeta^{\left(\ell \right)}\left(\sigma \;+\;it\right){|}^{2}dt∼\zeta^{\left(2\ell \right)}\left(2\sigma \right)T,\;\;as\;\;T\to \infty .$$

For $\sigma =\frac{1}{2}$, Ingham [16, p. 294, Theorem **A″**] has proved the following result on second moments of $\zeta^{\left(\ell \right)}\left(s\right)$.

Lemma 5 (Ingham) Let ℓ∈N be fixed. Then

$$\int_{0}^{T}{\left|\zeta^{\left(\ell \right)}\left(\frac{1}{2}+it\right)\right|}^{2}dt∼\frac{T}{2\ell +1}{\left(\log\frac{T}{2\pi }\right)}^{2\ell +1},\;\;as\;\;T\to \infty .$$

## 3 PROOF OF PROPOSITION 1

We will use the construction of Bondarenko and Seip in [9].

Let $\delta =\ell \cdot {\left(\ell +1\right)}^{-1}$. Given a positive number *y* and a positive integer *b*, define

$$P\left(y,b\right):=\prod_{p⩽y}p^{b-1}\;\;.$$

We will choose a number *x* and an integer *b* later to make $P\left(x,b\right)⩽\sqrt{T}$. Let M be the set of divisors of P(x,b) and $M_{\delta }$ be the set of divisors of $P(x^{\delta },b)$. Let $\;\;\bar{M_{\delta }}$ be the complement of $M_{\delta }$ in M. Note that both M and $M_{\delta }$ are divisor‐closed which means k|n,n∈M⇒k∈M and $k|n,n\in M_{\delta }\Rightarrow k\in M_{\delta }$. Define the function r:N→{0,1} to be the characteristic function of M, then

$$\begin{array}{c} |\sum_{mk=n⩽\sqrt{T}}\frac{r\left(m\right)\bar{r(n)}}{k}{\left(\log k\right)}^{\ell }|/\left(\sum_{n⩽\sqrt{T}}{\left|r\left(n\right)\right|}^{2}\right)=\frac{1}{\left|M\right|}\sum_{mk=n\in M}\frac{{\left(\log k\right)}^{\ell }}{k}=\frac{1}{\left|M\right|}\sum_{\begin{array}{c} n\in M\\ k|n \end{array}}\frac{{\left(\log k\right)}^{\ell }}{k}\;. \end{array}$$

As showed in [9],

$$\begin{array}{c} \frac{1}{\left|M\right|}\sum_{\begin{array}{c} n\in M\\ k|n \end{array}}\frac{1}{k}=\prod_{p⩽x}\left(1+\sum_{\nu =1}^{b-1}\left(1-\frac{\nu }{b}\right)p^{-\nu }\right)\;. \end{array}$$

Also in [9, p. 129, lines 3–9], it is proved that

(15) $$\begin{array}{c} \prod_{p⩽x}\left(1+\sum_{\nu =1}^{b-1}\left(1-\frac{\nu }{b}\right)p^{-\nu }\right)=\left(1+O\left(b^{-1}\right)+O\left(\frac{1}{\sqrt{x}\log x}\right)\right)\;e^{\gamma }\log x\;. \end{array}$$

Next, we split the sum into the following two parts:

$$\begin{array}{c} \frac{1}{\left|M\right|}\sum_{\begin{array}{c} n\in M\\ k|n \end{array}}\frac{1}{k}=\frac{1}{\left|M\right|}\sum_{\begin{array}{c} n\in M\\ k|n\\ k\in M_{\delta } \end{array}}\frac{1}{k}+\frac{1}{\left|M\right|}\sum_{\begin{array}{c} n\in M\\ k|n\\ k\in \bar{M_{\delta }} \end{array}}\frac{1}{k}\;. \end{array}$$

We will prove the following identity:

(16) $$\begin{array}{c} \frac{1}{\left|M\right|}\sum_{\begin{array}{c} n\in M\\ k|n\\ k\in M_{\delta } \end{array}}\frac{1}{k}=\prod_{p⩽x^{\delta }}\left(1+\sum_{\nu =1}^{b-1}\left(1-\frac{\nu }{b}\right)p^{-\nu }\right)\;. \end{array}$$

To see this, let *m* be the largest integer such that $p_{m}⩽x^{\delta }$ and let *w* be the largest integer such that $p_{w}⩽x$  ($p_{n}$ denotes the *n*th prime). Then we have

$$\begin{array}{cc} \sum_{\begin{array}{c} n\in M\\ k|n\\ k\in M_{\delta } \end{array}}\frac{1}{k} & =\sum_{k\in M_{\delta }}\frac{1}{k}\left(\sum_{\begin{array}{c} k|n\\ n\in M \end{array}}1\right)\\ & =\sum_{\alpha_{1}=0}^{b-1}\sum_{\alpha_{2}=0}^{b-1}\cdots \sum_{\alpha_{m}=0}^{b-1}\frac{1}{p_{1}^{\alpha_{1}}p_{2}^{\alpha_{2}}\cdots p_{m}^{\alpha_{m}}}\left(b-\alpha_{1}\right)\left(b-\alpha_{2}\right)\cdots \left(b-\alpha_{m}\right)\cdot b^{w-m}\\ & =b^{w-m}\cdot \prod_{n=1}^{m}\left(\sum_{\alpha_{n}=0}^{b-1}\frac{b-\alpha_{n}}{p_{n}^{\alpha_{n}}}\right)\\ & =b^{w}\prod_{n=1}^{m}\left(\sum_{\nu =0}^{b-1}\left(1-\frac{\nu }{b}\right)p_{n}^{-\nu }\right)\;. \end{array}$$

Note that $\left|M\right|=b^{w}$, then we immediately get (16). Now (15) together with (16) give that

(17) $$\begin{array}{c} \frac{1}{\left|M\right|}\sum_{\begin{array}{c} n\in M\\ k|n\\ k\in M_{\delta } \end{array}}\frac{1}{k}=\prod_{p⩽x^{\delta }}\left(1+\sum_{\nu =1}^{b-1}\left(1-\frac{\nu }{b}\right)p^{-\nu }\right)=\left(1+O\left(b^{-1}\right)+O\left(\frac{1}{\sqrt{x^{\delta }}\log x}\right)\right)\;e^{\gamma }\cdot \delta \cdot \log x\;, \end{array}$$

where we omit the term $\delta^{-1}$ inside the second big O(·) term since $1<\delta^{-1}⩽2$. Thus we obtain

$$\begin{array}{c} \frac{1}{\left|M\right|}\sum_{\begin{array}{c} n\in M\\ k|n\\ k\in \bar{M_{\delta }} \end{array}}\frac{1}{k}=\frac{1}{\left|M\right|}\sum_{\begin{array}{c} n\in M\\ k|n \end{array}}\frac{1}{k}-\frac{1}{\left|M\right|}\sum_{\begin{array}{c} n\in M\\ k|n\\ k\in M_{\delta } \end{array}}\frac{1}{k}=\left(1+O\left(b^{-1}\right)+O\left(\frac{1}{\sqrt{x^{\delta }}\log x}\right)\right)\;e^{\gamma }\left(1-\delta \right)\;\log x\;. \end{array}$$

By the definition of $\;\bar{M_{\delta }}$, if $k\in \;\;\bar{M_{\delta }}$, then logk⩾δlogx . So we have

$$\begin{array}{c} \frac{1}{\left|M\right|}\sum_{\begin{array}{c} n\in M\\ k|n \end{array}}\frac{{\left(\log k\right)}^{\ell }}{k}⩾\frac{1}{\left|M\right|}\sum_{\begin{array}{c} n\in M\\ k|n\\ k\in \bar{M_{\delta }} \end{array}}\frac{{\left(\log k\right)}^{\ell }}{k}⩾\left(\;1+O\left(b^{-1}\right)+O\left(\;\frac{1}{\sqrt{x^{\delta }}\log x}\;\right)\;\;\right)\;e^{\gamma }\left(1-\delta \right)\delta^{\ell }\;{\left(\log x\right)}^{\ell +1}. \end{array}$$

Now we set $x=\left(\log T\right)/\left(3\log_{2}T\right)$ and $b=[\log_{2}T]$. By the prime number theorem, $P\left(x,b\right)⩽\sqrt{T}$ when *T* is sufficiently large. Take the choices of x,b and $\delta =\ell \cdot {\left(\ell +1\right)}^{-1}$ into the above inequality, then we are done.□

## 4 PROOF OF THEOREM 1

Set $N=[T^{\frac{1}{2}}]$ and let $R\left(t\right):=\sum_{n⩽N}r\left(n\right)n^{-it}$. Define the moments as follows:

$$\begin{array}{cc} M_{1}\left(R,T\right) & :=\int_{T}^{2T}{\left|R\left(t\right)\right|}^{2}\Phi \left(\frac{t}{T}\right)dt,\\ M_{2}\left(R,T\right) & :=\int_{T}^{2T}{\left(-1\right)}^{\ell }\zeta^{\left(\ell \right)}\left(1+it\right){\left|R\left(t\right)\right|}^{2}\Phi \left(\frac{t}{T}\right)dt\;. \end{array}$$

As in [22], Φ:R→R denotes a smooth function, compactly supported in [1, 2], with 0⩽Φ(y)⩽1 for all *y*, and Φ(y)=1 for 5/4⩽y⩽7/4. Partial integration gives that $\hat{\Phi }\left(y\right)\ll_{\nu }{\left|y\right|}^{-\nu }$ for any positive integer ν.

Also in [22], Soundararajan proved that

(18) $$M_{1}\left(R,T\right)=T\hat{\Phi }\left(0\right)\left(1+O\left(T^{-1}\right)\right)\sum_{n⩽N}{\left|r\left(n\right)\right|}^{2}\;.$$

Since Φ is compactly supported in [1, 2], we deduce that

$$\begin{array}{c} \int_{T}^{2T}{\left|R\left(t\right)\right|}^{2}\sum_{k⩽T}\frac{{\left(\log k\right)}^{\ell }}{k^{1+it}}\Phi \left(\frac{t}{T}\right)dt=T\sum_{m,\;n⩽N}\sum_{k⩽T}\frac{r\left(m\right)\bar{r(n)}}{k}{\left(\log k\right)}^{\ell }\cdot \hat{\Phi }\left(T\cdot \log\frac{km}{n}\right)\;. \end{array}$$

Since $N⩽T^{\frac{1}{2}}$, for the off‐diagonal terms km≠n we have $\hat{\Phi }\left(T\log\left(km/n\right)\right)\ll T^{-2}$, by the rapid decay of $\hat{\Phi }$  (see [22, p. 471]). Thus the contribution of the off‐diagonal terms km≠n to the above summands can be bounded by

$$\begin{array}{c} \ll T{\left(\sum_{n⩽N}\left|r\left(n\right)\right|\right)}^{2}\cdot \sum_{k⩽T}\frac{{\left(\log k\right)}^{\ell }}{k}\cdot T^{-2}\ll T^{-1}{\left(\log T\right)}^{\ell +1}N\sum_{n⩽N}{\left|r\left(n\right)\right|}^{2}\;. \end{array}$$

Again, by $N=[T^{\frac{1}{2}}]$, we obtain

(19) $$\begin{array}{cc} \int_{T}^{2T}{\left|R\left(t\right)\right|}^{2}\sum_{k⩽T}\frac{{\left(\log k\right)}^{\ell }}{k^{1+it}}\Phi \left(\frac{t}{T}\right)dt= & \;\hat{\Phi }\left(0\right)T\sum_{mk=n⩽\sqrt{T}}\frac{r\left(m\right)\bar{r(n)}}{k}{\left(\log k\right)}^{\ell }\\ & +\;O\left(T^{-\frac{1}{2}}{\left(\log T\right)}^{\ell +1}\sum_{n⩽\sqrt{T}}{\left|r\left(n\right)\right|}^{2}\right)\;. \end{array}$$

By Lemma 1, we have the following approximation formula and the implied constant in the big O(·) term is absolute:

$${\left(-1\right)}^{\ell }\zeta^{\left(\ell \right)}\left(1+it\right)=\sum_{k⩽T}\frac{{\left(\log k\right)}^{\ell }}{k^{1+it}}+O\left(\frac{\ell !}{\varepsilon^{\ell }}\cdot T^{-1+\varepsilon }\right)\;,\;T⩽t⩽2T.$$

In the integral of $M_{2}\left(R,T\right)$, the big O(·) term above contributes at most

$$\begin{array}{c} \ll \int_{T}^{2T}\frac{\ell !}{\varepsilon^{\ell }}\cdot T^{-1+\varepsilon }\cdot |R\left(t\right){|}^{2}\Phi \left(\frac{t}{T}\right)dt\ll \frac{\ell !}{\varepsilon^{\ell }}\cdot T^{-1+\varepsilon }\cdot M_{1}\left(R,T\right)\;. \end{array}$$

Combining this with (19), we have

$$\begin{array}{cc} M_{2}\left(R,T\right) & =\hat{\Phi }\left(0\right)T\sum_{mk=n⩽\sqrt{T}}\frac{r\left(m\right)\bar{r(n)}}{k}{\left(\log k\right)}^{\ell }+\;O\left(T^{-\frac{1}{2}}{\left(\log T\right)}^{\ell +1}\sum_{n⩽\sqrt{T}}{\left|r\left(n\right)\right|}^{2}\right)\\ & \;+O\left(\frac{\ell !}{\varepsilon^{\ell }}\cdot T^{-1+\varepsilon }\right)\cdot M_{1}\left(R,T\right)\;. \end{array}$$

Finally, the above formula together with (18) gives that

$$\begin{array}{cc} \max_{T⩽t⩽2T}\left|\zeta^{\left(\ell \right)}\left(1+it\right)\right| & ⩾\frac{|M_{2}\left(R,T\right)|}{M_{1}\left(R,T\right)}\\ & ⩾\left(1+O(T^{-1})\right)\left|\sum_{mk=n⩽\sqrt{T}}\frac{r\left(m\right)\bar{r(n)}}{k}{\left(\log k\right)}^{\ell }\right|/\left(\sum_{n⩽\sqrt{T}}{\left|r\left(n\right)\right|}^{2}\right)\\ & \;+\;O\left(T^{-\frac{3}{2}}\;{\left(\log T\right)}^{\ell +1}\right)+O\left(\frac{\ell !}{\varepsilon^{\ell }}\cdot T^{-1+\varepsilon }\right)\;. \end{array}$$

Now let $\varepsilon ={\left(\log_{2}T\right)}^{-1}$. By Stirling's formula, if *T* is sufficiently large, then for all positive integers $\ell ⩽\left(\log T\right){\left(\log_{2}T\right)}^{-1}$, we have  $\ell !\cdot \varepsilon^{-\ell }\cdot T^{-1+\varepsilon }⩽{\left(\log_{2}T\right)}^{\ell }$. Other big O(·) terms can be easily bounded. Together with Proposition 1, we finish the proof of Theorem 1.□

## 5 PROOF OF THEOREM 2

### 5.1 Constructing the resonator

Given a set M of positive integers and a parameter *T*, we will construct a resonator R(t), following ideas from [1], [7], and [11]. Define

$$M_{j}:=\left[{\left(1+\frac{\log T}{T}\right)}^{j},{\left(1+\frac{\log T}{T}\right)}^{j+1}\right)\cap M\;\left(j⩾0\right).$$

Let J be the set of integers *j* such that $M_{j}\neq \emptyset$ and let $m_{j}$ be the minimum of $M_{j}$ for j∈J. We then set

$$M^{\prime }:=\{m_{j}:\;j\in J\}$$

and

$$r\left(m_{j}\right):=\sqrt{\sum_{m\in M_{j}}1}=\sqrt{|M_{j}|}$$

for every $m_{j}$ in $M^{\prime }$. Then the resonator R(t) is defined as follows:

(20) $$R\left(t\right):=\sum_{m\in M^{\prime }}\frac{r(m)}{m^{it}}\;.$$

By Cauchy's inequality, one has the following trivial estimates [11]:

$$R{\left(0\right)}^{2}⩽N\sum_{m\in M^{\prime }}r{\left(m\right)}^{2}⩽N\left|M\right|⩽N^{2}\;.$$

As in [7] , set $\Phi \left(t\right):=e^{-t^{2}/2}$. Its Fourier transform satisfies $\hat{\Phi }\left(\xi \right)=\sqrt{2\pi }\Phi \left(\xi \right)$.

Replacing *T* by T/logT in [8, Lemma 5], gives that

(21) $$\int_{-\infty }^{\infty }{\left|R\left(t\right)\right|}^{2}\Phi \left(\frac{t\log T}{T}\right)dt\ll \frac{T|M|}{\log T}\;.$$

### 5.2 The proof

Let $\sigma \in [\frac{1}{2},1)$. Choose κ∈(0,1−β) and set $N:=[T^{\kappa }]$. Fix ε>0 such that κ+4ε<1.

As in [8], choose

$$K\left(t\right):=\frac{\sin^{2}\left(\left(\varepsilon \log T\right)t\right)}{\left(\varepsilon \log T\right)t^{2}},$$

which has Fourier transform

(22) $$\hat{K}\left(\xi \right)=\pi \max\left(\left(1-\frac{\left|\xi \right|}{2\varepsilon \log T}\right),0\right).$$

Define

$$\begin{array}{cc} Z_{\sigma }\left(t,y\right) & :=F_{\ell }\left(\sigma +it+iy\right)F_{\ell }\left(\sigma -it+iy\right)K\left(y\right)\;,\\ I(T) & :=\int_{|t|⩾2}{\left|R\left(t\right)\right|}^{2}\Phi \left(\frac{t\log T}{T}\right)\int_{-\infty }^{\infty }Z_{\sigma }\left(t,y\right)dydt\;. \end{array}$$

Following [8] and [11], we will show that the integral on $2T^{\beta }⩽\left|t\right|⩽\frac{T}{2}$ and $\left|y\right|⩽\frac{\left|t\right|}{2}$ gives the main term for I(T). We will frequently use the following trivial estimates (Lemma 2):

(23) $$|F_{\ell }\left(\sigma \pm it+iy\right)|\ll 1+|\zeta^{\left(\ell \right)}{\left(\sigma \pm it+iy\right)|\ll (1+|t|+|y|)}^{\frac{3}{10}}.$$

A simple computation gives

$$\int_{2⩽\left|t\right|⩽2T^{\beta }}\int_{|y|>T^{\beta }}Z_{\sigma }\left(t,y\right)dydt\ll \int_{\left|t\right|⩽2T^{\beta }}\int_{|y|>T^{\beta }}{\left(1+|t|+|y|\right)}^{\frac{3}{5}}\frac{1}{\left(|t\right|+{\left|y|\right)}^{2}}dydt\ll {\left(T^{\beta }\right)}^{\frac{3}{5}}\;.$$

Note that

$$\begin{array}{cc} & F_{\ell }\left(\sigma +it+iy\right)F_{\ell }\left(\sigma -it+iy\right)\\ & \;\ll \left(1+|\zeta^{\left(\ell \right)}\left(\sigma +it+iy\right)|\right)\left(1+|\zeta^{\left(\ell \right)}\left(\sigma -it+iy\right)|\right)\\ & \;\ll 1+|\zeta^{\left(\ell \right)}\left(\sigma +it+iy\right)|+|\zeta^{\left(\ell \right)}\left(\sigma -it+iy\right)|+|\zeta^{\left(\ell \right)}{\left(\sigma +it+iy\right)|}^{2}+{\left|\zeta^{\left(\ell \right)}\left(\sigma -it+iy\right)\right|}^{2}. \end{array}$$

Thus

$$\begin{array}{cc} \int_{2⩽\left|t\right|⩽2T^{\beta }}\int_{-\infty }^{\infty }Z_{\sigma }\left(t,y\right)dydt & \ll T^{\beta }+\int_{2⩽\left|t\right|⩽2T^{\beta }}\int_{\left|y\right|⩽T^{\beta }}Z_{\sigma }\left(t,y\right)dydt\\ & \ll T^{\beta }+\int_{-3T^{\beta }}^{3T^{\beta }}|\zeta^{\left(\ell \right)}\left(\sigma +it\right)|dt+\int_{-3T^{\beta }}^{3T^{\beta }}{\left|\zeta^{\left(\ell \right)}\left(\sigma +it\right)\right|}^{2}dt\\ & \ll T^{\beta }\cdot {\left(\log T\right)}^{2\ell +1}, \end{array}$$

where the last step follows from Lemmas 4 and 5 and the Cauchy–Schwarz inequality. Trivially, by |R(t)|⩽R(0) and Φ(·)⩽1,

$$\int_{2⩽\left|t\right|⩽2T^{\beta }}{\left|R\left(t\right)\right|}^{2}\Phi \left(\frac{t\log T}{T}\right)\int_{-\infty }^{\infty }Z_{\sigma }\left(t,y\right)dydt\ll R{\left(0\right)}^{2}T^{\beta }\cdot {\left(\log T\right)}^{2\ell +1}\ll \left|M\right|T^{\beta +\kappa }{\left(\log T\right)}^{2\ell +1}.$$

The fast decay of Φ and (23) give that

$$\int_{|t|>\frac{T}{2}}{\left|R\left(t\right)\right|}^{2}\Phi \left(\frac{t\log T}{T}\right)\int_{-\infty }^{\infty }Z_{\sigma }\left(t,y\right)dydt\ll T^{\kappa +4}e^{-\frac{1}{16}{\left(\log T\right)}^{2}}\cdot \left|M\right|\ll o\left(1\right)\cdot |M|\;.$$

Using (21) and (23), one can compute

$$\int_{2T^{\beta }⩽\left|t\right|⩽\frac{T}{2}}{\left|R\left(t\right)\right|}^{2}\Phi \left(\frac{t\log T}{T}\right)\int_{|y|>\frac{\left|t\right|}{2}}Z_{\sigma }\left(t,y\right)dydt\ll \frac{T^{1-\frac{2}{5}\beta }}{{\left(\log T\right)}^{2}}\cdot \left|M\right|\;.$$

Combining the above estimates, one gets

$$\begin{array}{cc} I\left(T\right)=\int_{2T^{\beta }⩽\left|t\right|⩽\frac{T}{2}}{\left|R\left(t\right)\right|}^{2}\Phi \left(\frac{t\log T}{T}\right)\int_{\left|y\right|⩽\frac{\left|t\right|}{2}}Z_{\sigma }\left(t,y\right)dydt & +\left|M\right|\cdot O\left(T^{\beta +\kappa }{\left(\log T\right)}^{2\ell +1}\right)\\ & +\left|M\right|\cdot O\left(\frac{T^{1-\frac{2}{5}\beta }}{{\left(\log T\right)}^{2}}\right)\;. \end{array}$$

Note that $2T^{\beta }⩽\left|t\right|⩽\frac{T}{2}$ and $\left|y\right|⩽\frac{\left|t\right|}{2}$ give $T^{\beta }⩽\left|t\pm y\right|⩽T$.  Again, by (21)

(24) $$\begin{array}{c} I\left(T\right)\ll \frac{T|M|}{\log T}\cdot \max_{T^{\beta }⩽t⩽T}{\left|F_{\ell }\left(\sigma +it\right)\right|}^{2}+\left|M\right|\cdot O\left(T^{\beta +\kappa }{\left(\log T\right)}^{2\ell +1}\right)+\left|M\right|\cdot O\left(\frac{T^{1-\frac{2}{5}\beta }}{{\left(\log T\right)}^{2}}\right). \end{array}$$

Next, let

(25) $$G_{\sigma }\left(t\right):=\sum_{m,n⩾1}\frac{\hat{K}\left(\log nm\right)}{n^{\sigma +it}\cdot m^{\sigma -it}}a_{\ell }\left(n\right)a_{\ell }\left(m\right)$$

and set

$$\begin{array}{cc} I_{1}\left(T\right) & :=\int_{|t|⩾2}G_{\sigma }\left(t\right){\left|R\left(t\right)\right|}^{2}\Phi \left(\frac{t\log T}{T}\right)dt\\ I_{2}\left(T\right) & :=-2\pi \ell !\int_{|t|⩾2}\Delta^{+}\cdot {\left|R\left(t\right)\right|}^{2}\Phi \left(\frac{t\log T}{T}\right)dt\\ I_{3}\left(T\right) & :=-2\pi \ell !\int_{|t|⩾2}\Delta^{-}\cdot {\left|R\left(t\right)\right|}^{2}\Phi \left(\frac{t\log T}{T}\right)dt. \end{array}$$

By the convolution formula (10), one obtains

$$I\left(T\right)=I_{1}\left(T\right)+I_{2}\left(T\right)+I_{3}\left(T\right).$$

We will bound $I_{2}\left(T\right),I_{3}\left(T\right)$ as follows:

(26) $$\begin{array}{c} |I_{2}\left(T\right)|+|I_{3}\left(T\right)|\ll |M|\cdot \frac{T^{\kappa +\frac{5}{4}\varepsilon }}{\log T}\;. \end{array}$$

By Cauchy's integral for derivatives and the explicit expression for *K*, we have the following estimates for all 0⩽n⩽l:

(27) $$\begin{array}{c} {\left(\frac{d}{dz}\right)}^{n}{K\left(i\sigma -iz\right)|}_{z=1-it}\ll \max_{\left|\alpha \right|=\frac{1}{8}}|K\left(i\sigma -i(1-it)+\alpha \right)|\ll \frac{T^{\frac{5}{4}\varepsilon }}{\log T\cdot {\left|t\right|}^{2}},\;\forall \left|t\right|⩾2, \end{array}$$

where the implied constants depend on ε and ℓ only.

And trivially, for all 0⩽m⩽l, one has

(28) $$\begin{array}{c} {\left(\frac{d}{dz}\right)}^{m}F_{\ell }{\left(z-it\right)|}_{z=1-it}\ll 1+|\zeta^{\left(\ell +m\right)}{\left(1-2it\right)|\ll |t|}^{\frac{1}{8}},\;\forall \left|t\right|⩾2, \end{array}$$

where the implied constants depend only on ℓ.

Note that there are finitely many non‐negative integer pairs (m,n) satisfying m+n=ℓ, so

(29) $$\begin{array}{c} I_{3}\left(T\right)\ll R{\left(0\right)}^{2}\int_{|t|⩾2}\frac{T^{\frac{5}{4}\varepsilon }\cdot {\left|t\right|}^{\frac{1}{8}}}{\log T\cdot {\left|t\right|}^{2}}dt\ll (T^{\kappa }\cdot \left|M|\right)\cdot \frac{T^{\frac{5}{4}\varepsilon }}{\log T}\int_{|t|⩾2}\frac{{\left|t\right|}^{\frac{1}{8}}}{{\left|t\right|}^{2}}dt\ll \left|M\right|\cdot \frac{T^{\kappa +\frac{5}{4}\varepsilon }}{\log T}\;. \end{array}$$

Proceed similarly for $I_{2}\left(T\right)$, so we get (26).

Next, in order to relate $I_{1}\left(T\right)$ to the GCD sums, we would like to use Fourier transform on the whole real line. So set

$$\begin{array}{cc} \tilde{I_{1}}\left(T\right) & :=\int_{-\infty }^{\infty }G_{\sigma }\left(t\right){\left|R\left(t\right)\right|}^{2}\Phi \left(\frac{t\log T}{T}\right)dt\;. \end{array}$$

By (22), $\hat{K}\left(\log nm\right)=0$ if $mn⩾T^{2\varepsilon }$. Clearly, $a_{l}\left(n\right)/n^{\sigma }\ll 1$. So one can get

$$\begin{array}{cc} \int_{|t|⩽2}G_{\sigma }\left(t\right){\left|R\left(t\right)\right|}^{2}\Phi \left(\frac{t\log T}{T}\right)dt & =\int_{|t|⩽2}\left(\sum_{m,n⩾1}\frac{\hat{K}\left(\log nm\right)}{n^{\sigma +it}\cdot m^{\sigma -it}}a_{\ell }\left(n\right)a_{\ell }\left(m\right)\right){\left|R\left(t\right)\right|}^{2}\Phi \left(\frac{t\log T}{T}\right)dt\\ & \ll R{\left(0\right)}^{2}\sum_{m,n⩾1}\frac{\hat{K}\left(\log nm\right)}{n^{\sigma }\cdot m^{\sigma }}a_{\ell }\left(n\right)a_{\ell }\left(m\right)\\ & \ll (T^{\kappa }\cdot \left|M|\right)\cdot \sum_{mn⩽T^{2\varepsilon }}1\\ & \ll T^{\kappa +4\varepsilon }\cdot \left|M\right|\;. \end{array}$$

We obtain $I_{1}\left(T\right)=\tilde{I_{1}}\left(T\right)+\left|M\right|\cdot O\left(T^{\kappa +4\varepsilon }\right)$. Thus we have

(30) $$\begin{array}{cc} \tilde{I_{1}}\left(T\right)\ll \frac{T|M|}{\log T}\cdot \max_{T^{\beta }⩽t⩽T}{\left|F_{\ell }\left(\sigma +it\right)\right|}^{2}+\left|M\right|\cdot O\left(T^{\kappa +4\varepsilon }\right) & +\left|M\right|\cdot O\left(T^{\beta +\kappa }{\left(\log T\right)}^{2\ell +1}\right)\\ & +\left|M\right|\cdot O\left(\frac{T^{1-\frac{2}{5}\beta }}{{\left(\log T\right)}^{2}}\right). \end{array}$$

We compute the integral $\tilde{I_{1}}\left(T\right)$ by expanding the product of the resonator and the infinite series of $G_{\sigma }\left(t\right)$, and then integrate term by term, as in [7, p. 1699]. Using the fact $a_{\ell }\left(k\right)⩾1$ for every *k* and $\hat{K}\left(\log jk\right)⩾\pi /2$ if $jk⩽T^{\varepsilon }$, one gets

$$\begin{array}{cc} \tilde{I_{1}}\left(T\right) & =\frac{T\sqrt{2\pi }}{\log T}\sum_{m,n\in M^{\prime }}r\left(m\right)r\left(n\right)\sum_{j,k⩾1}a_{\ell }\left(j\right)a_{\ell }\left(k\right)\frac{\hat{K}\left(\log jk\right)}{{\left(jk\right)}^{\sigma }}\Phi \left(\frac{T}{\log T}\log\frac{mj}{nk}\right)\\ & ⩾\frac{T\sqrt{2\pi }}{\log T}\sum_{m,n\in M^{\prime }}r\left(m\right)r\left(n\right)\sum_{j,k⩾1}\frac{\hat{K}\left(\log jk\right)}{{\left(jk\right)}^{\sigma }}\Phi \left(\frac{T}{\log T}\log\frac{mj}{nk}\right)\\ & \gg \frac{T}{\log T}\sum_{1⩽jk⩽T^{\varepsilon }}\frac{1}{{\left(jk\right)}^{\sigma }}\sum_{m,n\in M^{\prime }}r\left(m\right)r\left(n\right)\Phi \left(\frac{T}{\log T}\log\frac{mj}{nk}\right)\;. \end{array}$$

Next, proceed as in [11, p. 127–128] (following ideas from [7]),

(31) $$\begin{array}{c} \tilde{I_{1}}\left(T\right)\gg \frac{T}{\log T}\sum_{\begin{array}{c} m,n\in M\\ \frac{\left[m,n\right]}{\left(m,n\right)}⩽T^{\varepsilon } \end{array}}\frac{{\left(m,n\right)}^{\sigma }}{{\left[m,n\right]}^{\sigma }}\gg \frac{T}{\log T}\left(S_{\sigma }\left(M\right)-T^{\varepsilon (\frac{1}{3}-\sigma )}\cdot S_{\frac{1}{3}}\left(M\right)\right)\;. \end{array}$$

Combining (30) with (31), we have

(32) $$\begin{array}{cc} & \max_{T^{\beta }⩽t⩽T}{\left|F_{\ell }\left(\sigma +it\right)\right|}^{2}+O\left(T^{\beta +\kappa -1}{\left(\log T\right)}^{2\ell +2}\right)+O\left(T^{\kappa +4\varepsilon -1}\log T\right)+O\left(\frac{T^{-\frac{2}{5}\beta }}{\log T}\right)\\ & \;\gg \frac{S_{\sigma }\left(M\right)}{\left|M\right|}-T^{\varepsilon (\frac{1}{3}-\sigma )}\cdot \frac{S_{1/3}\left(M\right)}{\left|M\right|}\;. \end{array}$$

Next, we will consider the two cases $\sigma =\frac{1}{2}$ and $\sigma \in (\frac{1}{2},1)$ separately.

**Case 1**: $\sigma =\frac{1}{2}$.

In this case, let M be the set in (4) with |M|=N. Recall that $N=[T^{\kappa }]$, so

(33) $$\begin{array}{c} \frac{S_{1/2}\left(M\right)}{\left|M\right|}\gg exp\{\left(2\sqrt{2\;\kappa }+o\left(1\right)\right)\sqrt{\frac{\log T\;\log_{3}T}{\log_{2}T}}\}\;. \end{array}$$

Also, in [11, p. 128], de la Bretèche and Tenenbaum showed that for this set M,

(34) $$\begin{array}{c} \frac{S_{1/3}\left(M\right)}{\left|M\right|}\ll exp\{y_{M}^{\frac{2}{3}}\},\;where\;y_{M}\ll {\left(\log T\right)}^{\frac{6}{5}}. \end{array}$$

So the second term on the right‐hand side of (32) is *o*(1). And clearly, the big O(·) terms in (32) can be ignored. Thus

(35) $$\begin{array}{c} \max_{T^{\beta }⩽t⩽T}\left|\zeta^{\left(\ell \right)}\left(\frac{1}{2}+it\right)\right|\gg \max_{T^{\beta }⩽t⩽T}\left|F_{\ell }\left(\frac{1}{2}+it\right)\right|+O\left(1\right)\gg exp\{\left(\sqrt{2\;\kappa }+o\left(1\right)\right)\sqrt{\frac{\log T\;\log_{3}T}{\log_{2}T}}\}\;. \end{array}$$

**Case 2**: $\sigma \in (\frac{1}{2},1)$.

In this case, let M be the set in (6) with |M|=N. Again, $N=[T^{\kappa }]$, so

(36) $$\begin{array}{c} \frac{S_{\sigma }\left(M\right)}{\left|M\right|}\gg exp\{\frac{\tilde{c}}{1-\sigma }\cdot \frac{{\left(\log N\right)}^{1-\sigma }}{{\left(\log_{2}N\right)}^{\sigma }}\}\gg exp\{\frac{\tilde{c}\cdot \kappa^{1-\sigma }}{1-\sigma }\cdot \frac{{\left(\log T\right)}^{1-\sigma }}{{\left(\log_{2}T\right)}^{\sigma }}\}. \end{array}$$

Similarly as (31), we have

$$\begin{array}{c} \tilde{I_{1}}\left(T\right)\gg \frac{T}{\log T}\left(S_{\sigma }\left(M\right)-T^{\varepsilon (\frac{1}{2}-\sigma )}\cdot S_{\frac{1}{2}}\left(M\right)\right)\;. \end{array}$$

And by (4), we can get the following estimates:

$$\begin{array}{c} T^{\varepsilon (\frac{1}{2}-\sigma )}\cdot \frac{S_{\frac{1}{2}}\left(M\right)}{\left|M\right|}\ll T^{\varepsilon (\frac{1}{2}-\sigma )}exp\{\left(2\sqrt{2}+o\left(1\right)\right)\sqrt{\frac{\kappa \log T\;\log_{3}T}{\log_{2}T}}\}=o\left(1\right). \end{array}$$

Hence,

(37) $$\begin{array}{c} \max_{T^{\beta }⩽t⩽T}\left|\zeta^{\left(\ell \right)}\left(\sigma +it\right)\right|\gg \max_{T^{\beta }⩽t⩽T}\left|F_{\ell }\left(\sigma +it\right)\right|+O\left(1\right)\gg exp\{\frac{\tilde{c}\cdot \kappa^{1-\sigma }}{2(1-\sigma )}\cdot \frac{{\left(\log T\right)}^{1-\sigma }}{{\left(\log_{2}T\right)}^{\sigma }}\}. \end{array}$$

Make κ slightly larger in the beginning then one can get (*B*).□

## 6 PROOF OF PROPOSITION 2

The idea of the proof is basically the same as in the proof of Proposition 1. The new ingredient is Gál's identity. In this section, in order to avoid confusion about notations, we use the notation (m⊗n) for the ordered pair of *m* and *n*.

Let $\tilde{P}\left(r,b\right)=p_{1}^{b-1}\cdot \text{…}\cdot p_{r}^{b-1}$ , where $p_{n}$ denotes the *n*th prime. Define M to be the set of divisors of $\tilde{P}\left(r,b\right)$, then $\left|M\right|=b^{r}$. By Gál's identity [13],

$$\sum_{m,n\in M}\frac{\left(m,n\right)}{\left[m,n\right]}=\prod_{i⩽r}\left(b+2\sum_{\nu =1}^{b-1}\frac{b-\nu }{p_{i}^{\nu }}\right)\;.$$

Let r=[logN/loglogN], then $p_{r}∼\log N$ by the prime number theorem. Let *b* be the integer satisfying that

$$b^{r}⩽N<{\left(b+1\right)}^{r},$$

then $b^{r}∼N$, as N→∞. Choose a set $M^{\prime }\subset N$ such that $M\subset M^{\prime }$ and $|M^{\prime }|=N$.

Following Lewko–Radziwiłł  in [19], we use Gál's identity for the GCD sum and then split the product into two parts:

(38) $$\begin{array}{cc} \sum_{m,n\in M}\frac{\left(m,n\right)}{\left[m,n\right]} & =b^{r}\prod_{i⩽r}\left(1+2\sum_{v=1}^{b-1}\frac{1}{p_{i}^{v}}\cdot \left(1-\frac{v}{b}\right)\right)\\ & ⩾\left(1+o\left(1\right)\right)N\prod_{i⩽r}{\left(1-\frac{1}{p_{i}}\right)}^{-2}\times \prod_{i⩽r}\left(1+2\sum_{v=1}^{b-1}\frac{1}{p_{i}^{v}}\cdot \left(1-\frac{v}{b}\right)\right){\left(1-\frac{1}{p_{i}}\right)}^{2}\;. \end{array}$$

By Mertens' theorem, the first product is asymptotically equal to ${\left(e^{\gamma }\log p_{r}\right)}^{2}∼{\left(e^{\gamma }\log\log N\right)}^{2}$ as N→∞. The second product converges as N→∞ to

$$\prod_{p}\left(1+2\sum_{v=1}^{\infty }\frac{1}{p^{v}}\right){\left(1-\frac{1}{p}\right)}^{2}=\frac{6}{\pi^{2}}.$$

Next, let $\;\;\delta =\ell \cdot {\left(2\ell +1\right)}^{-1}$ and define the sets $M_{\delta }^{\left(1\right)},\;\;M_{\delta }^{\left(2\right)}$ as follows:

$M_{\delta }^{\left(1\right)}:=\left\{\left(m⊗n\right)\in M\times M\right|\;\forall i>r^{\delta },\;\;\alpha_{i}=\min\left\{\alpha_{i},\beta_{i}\right\}$,   where *m* and *n* have prime factorizations as     $m=p_{1}^{\alpha_{1}}p_{2}^{\alpha_{2}}\cdots p_{r}^{\alpha_{r}},\;\;\;and\;\;\;n=p_{1}^{\beta_{1}}p_{2}^{\beta_{2}}\cdots p_{r}^{\beta_{r}}\}\;$.

$M_{\delta }^{\left(2\right)}:=\left\{\left(m⊗n\right)\in M\times M\right|\;\forall i>r^{\delta },\;\;\beta_{i}=\min\left\{\alpha_{i},\beta_{i}\right\}$,   where *m* and *n* have prime factorizations as     $m=p_{1}^{\alpha_{1}}p_{2}^{\alpha_{2}}\cdots p_{r}^{\alpha_{r}},\;\;\;and\;\;\;n=p_{1}^{\beta_{1}}p_{2}^{\beta_{2}}\cdots p_{r}^{\beta_{r}}\}\;$.

Then define $M_{\delta }$ to be the union of the above two sets and $\;\;\bar{M_{\delta }}$ to be the complement of $M_{\delta }$ in M×M:

$$M_{\delta }:=M_{\delta }^{\left(1\right)}⋃M_{\delta }^{\left(2\right)}\;,\;\;\;\bar{M_{\delta }}:=\left(M\times M\right)\setminus M_{\delta }\;.$$

Now we split the GCD sum into two parts:

(39) $$\begin{array}{c} \sum_{m,n\in M}\frac{\left(m,n\right)}{\left[m,n\right]}=\sum_{\left(m⊗n\right)\in M_{\delta }}\frac{\left(m,n\right)}{\left[m,n\right]}+\sum_{\left(m⊗n\right)\in \bar{M_{\delta }}}\frac{\left(m,n\right)}{\left[m,n\right]}\;. \end{array}$$

By symmetry, we have

(40) $$\begin{array}{c} \sum_{\left(m⊗n\right)\in M_{\delta }}\frac{\left(m,n\right)}{\left[m,n\right]}⩽2\sum_{\left(m⊗n\right)\in M_{\delta }^{\left(1\right)}}\frac{\left(m,n\right)}{\left[m,n\right]}\;. \end{array}$$

By the definition of $M_{\delta }^{\left(1\right)}$ and Gál's identity, we have

$$\begin{array}{cc} \sum_{\left(m⊗n\right)\in M_{\delta }^{\left(1\right)}}\frac{\left(m,n\right)}{\left[m,n\right]} & =\sum_{\begin{array}{c} i⩽r^{\delta }\\ 0⩽\alpha_{i}⩽b-1\\ 0⩽\beta_{i}⩽b-1 \end{array}}\;\;\;\;\;\;\sum_{\begin{array}{c} r^{\delta }<i⩽r\\ 0⩽\alpha_{i}⩽\beta_{i}⩽b-1 \end{array}}\prod_{i⩽r^{\delta }}p_{i}^{-|\alpha_{i}-\beta_{i}|}\prod_{r^{\delta }<i⩽r}\frac{1}{p_{i}^{\beta_{i}-\alpha_{i}}}\\ & =\prod_{i⩽r^{\delta }}\left(b+2\sum_{\nu =1}^{b-1}\frac{b-\nu }{p_{i}^{\nu }}\right)\;\;\;\cdot \prod_{r^{\delta }<i⩽r}\left(\sum_{x_{i}=0}^{b-1}\frac{b-x_{i}}{p_{i}^{x_{i}}}\right)\\ & =\prod_{i⩽r^{\delta }}\left(b+2\sum_{\nu =1}^{b-1}\frac{b-\nu }{p_{i}^{\nu }}\right)\;\;\;\cdot \prod_{r^{\delta }<i⩽r}\;\left({\left(\frac{1}{p_{i}}\right)}^{b+1}-\frac{b+1}{p_{i}}+b\right)\cdot {\left(1-\frac{1}{p_{i}}\right)}^{-2}\\ & =b^{r}\prod_{i⩽r^{\delta }}\left(1+2\sum_{\nu =1}^{b-1}\left(1-\frac{\nu }{b}\right)p_{i}^{-\nu }\right)\;\;\;\cdot \prod_{r^{\delta }<i⩽r}\;\left(\frac{1}{b}\cdot {\left(\frac{1}{p_{i}}\right)}^{b+1}-\frac{1+\frac{1}{b}}{p_{i}}+1\right)\\ & \;\cdot {\left(1-\frac{1}{p_{i}}\right)}^{-2}\\ & =b^{r}\prod_{i⩽r^{\delta }}\left(1+2\sum_{\nu =1}^{b-1}\left(1-\frac{\nu }{b}\right)p_{i}^{-\nu }\right){\left(1-\frac{1}{p_{i}}\right)}^{2}\\ & \;\times \prod_{r^{\delta }<i⩽r}\;\left(\frac{1}{b}\cdot {\left(\frac{1}{p_{i}}\right)}^{b+1}-\frac{1+\frac{1}{b}}{p_{i}}+1\right)\times \prod_{i⩽r}\;{\left(1-\frac{1}{p_{i}}\right)}^{-2}. \end{array}$$

Again, we have $b^{r}∼N$, the first product converges to $6/\pi^{2}$, and the third product is asymptotically equal to ${\left(e^{\gamma }\log p_{r}\right)}^{2}∼{\left(e^{\gamma }\log\log N\right)}^{2}$ as N→∞.

For the second product, it can be bounded as

$$\begin{array}{c} \prod_{r^{\delta }<i⩽r}\;\left(\frac{1}{b}\cdot {\left(\frac{1}{p_{i}}\right)}^{b+1}-\frac{1+\frac{1}{b}}{p_{i}}+1\right)⩽\prod_{r^{\delta }<i⩽r}\;\left(1-\frac{1}{p_{i}}\right)\;. \end{array}$$

And by Mertens' theorem and the prime number theorem, we have

$$\begin{array}{c} \lim_{r\to \infty }\prod_{r^{\delta }<i⩽r}\;\left(1-\frac{1}{p_{i}}\right)=\delta \;. \end{array}$$

As a result, we obtain that

$$\begin{array}{c} \sum_{\left(m⊗n\right)\in M_{\delta }^{\left(1\right)}}\frac{\left(m,n\right)}{\left[m,n\right]}⩽\left(\delta +o(1)\right)N\cdot \frac{6}{\pi^{2}}\cdot {\left(e^{\gamma }\log\log N\right)}^{2}\;. \end{array}$$

Hence by (38), (39), and (40), we get

$$\begin{array}{c} \sum_{\left(m⊗n\right)\in \bar{M_{\delta }}}\frac{\left(m,n\right)}{\left[m,n\right]}⩾\left(1-2\delta +o(1)\right)N\cdot \frac{6}{\pi^{2}}\cdot {\left(e^{\gamma }\log\log N\right)}^{2}\;. \end{array}$$

By the construction of $\;\;\;\bar{M_{\delta }}$,    if $\left(m⊗n\right)\in \bar{M_{\delta }}$, then

$$\begin{array}{c} \log\left(\frac{m}{\left(m,n\right)}\right)⩾\left(\delta +o(1)\right)\cdot \log\log N\;,\;\;\log\left(\frac{n}{\left(m,n\right)}\right)⩾\left(\delta +o(1)\right)\cdot \log\log N\;. \end{array}$$

Thus

$$\begin{array}{cc} & \sum_{m,n\in M^{\prime }}\frac{\left(m,n\right)}{\left[m,n\right]}\log^{\ell }\left(\frac{m}{\left(m,n\right)}\right)\log^{\ell }\left(\frac{n}{\left(m,n\right)}\right)\\ & \;⩾\sum_{m,n\in M}\frac{\left(m,n\right)}{\left[m,n\right]}\log^{\ell }\left(\frac{m}{\left(m,n\right)}\right)\log^{\ell }\left(\frac{n}{\left(m,n\right)}\right)\\ & \;⩾\sum_{\left(m⊗n\right)\in \bar{M_{\delta }}}\frac{\left(m,n\right)}{\left[m,n\right]}\log^{\ell }\left(\frac{m}{\left(m,n\right)}\right)\log^{\ell }\left(\frac{n}{\left(m,n\right)}\right)\\ & \;⩾\left(\left(1-2\delta \right)\;\delta^{2\ell }+o\left(1\right)\right)N\cdot \frac{6}{\pi^{2}}\cdot e^{2\gamma }\cdot {\left(\log\log N\right)}^{2+2\ell }. \end{array}$$

By our choice of $\;\;\delta =\ell \cdot {\left(2\ell +1\right)}^{-1}$, we are done.□

## 7 A SHORT PROOF FOR A WEAKER RESULT

One can use the method of Bohr–Landau (see [23, Theorem 8.5]) to prove the weaker result that $\zeta^{\left(\ell \right)}\left(1+it\right)=\Omega \left({\left(\log_{2}t\right)}^{\ell +1}\right)$, when ℓ∈N is fixed.

Write s=σ+it. When σ>1,

$${\left(-1\right)}^{\ell }\zeta^{\left(\ell \right)}\left(s\right)=\sum_{n=2}^{\infty }\frac{{\left(\log n\right)}^{\ell }}{n^{\sigma +it}}=\sum_{n=2}^{N}\frac{{\left(\log n\right)}^{\ell }}{n^{\sigma +it}}+\sum_{n=N+1}^{\infty }\frac{{\left(\log n\right)}^{\ell }}{n^{\sigma +it}}.$$

For given positive integers *N* and *q*, by Dirichlet's theorem, there exists $t\in [1,\;q^{N}]$, such that cos(tlogn)⩾cos(2π/q) for all integers n∈[1,N]. Hence

$$\begin{array}{cc} |\zeta^{\left(\ell \right)}\left(s\right)| & ⩾\sum_{n=2}^{N}\frac{{\left(\log n\right)}^{\ell }}{n^{\sigma }}\cos\left(t\log n\right)\;\;-\;\;\sum_{n=N+1}^{\infty }\frac{{\left(\log n\right)}^{\ell }}{n^{\sigma }}\\ & ⩾\cos\left(\frac{2\pi }{q}\right)\cdot \sum_{n=2}^{N}\frac{{\left(\log n\right)}^{\ell }}{n^{\sigma }}\;\;-\;\;\sum_{n=N+1}^{\infty }\frac{{\left(\log n\right)}^{\ell }}{n^{\sigma }}\\ & ⩾\cos\left(\frac{2\pi }{q}\right)\cdot \sum_{n=2}^{\infty }\frac{{\left(\log n\right)}^{\ell }}{n^{\sigma }}\;\;-\;\;2\sum_{n=N+1}^{\infty }\frac{{\left(\log n\right)}^{\ell }}{n^{\sigma }}. \end{array}$$

Take q=8 to get

(41) $$\begin{array}{cc} |\zeta^{\left(\ell \right)}\left(s\right)| & ⩾\cos\left(\frac{2\pi }{8}\right)\cdot \sum_{n=2}^{\infty }\frac{{\left(\log n\right)}^{\ell }}{n^{\sigma }}\;\;-\;\;2\sum_{n=N+1}^{\infty }\frac{{\left(\log n\right)}^{\ell }}{n^{\sigma }},\;N\;\log8>\log t\;. \end{array}$$

One can compute that

(42) $$\begin{array}{c} \sum_{n=2}^{\infty }\frac{{\left(\log n\right)}^{\ell }}{n^{\sigma }}>O_{\ell }\left(1\right)+\int_{1}^{\infty }\frac{{\left(\log x\right)}^{\ell }}{x^{\sigma }}dx>O_{\ell }\left(1\right)+\frac{\ell !}{{\left(\sigma -1\right)}^{\ell +1}}\;, \end{array}$$

and for large *N* that

(43) $$\begin{array}{c} \sum_{n=N+1}^{\infty }\frac{{\left(\log n\right)}^{\ell }}{n^{\sigma }}<\int_{N}^{\infty }\frac{{\left(\log x\right)}^{\ell }}{x^{\sigma }}dx⩽\left(\ell +1\right)\cdot {\left(\log N^{\sigma -1}\right)}^{\ell }\cdot N^{1-\sigma }\cdot \frac{\ell !}{{\left(\sigma -1\right)}^{\ell +1}}\;. \end{array}$$

Now fix a positive constant *A* (only depending on ℓ) such that $\left(\ell +1\right)A^{\ell }\cdot e^{-A}<1/12$ and let σ−1=A/logN. Combining with (41) gives that

(44) $$\begin{array}{c} |\zeta^{\left(\ell \right)}\left(s\right)|>\frac{\ell !}{{\left(\sigma -1\right)}^{\ell +1}}\cdot \left(\frac{1}{2}-2\cdot \frac{1}{12}\right)>\frac{\ell !}{3}\cdot \frac{{\left(\log N\right)}^{\ell +1}}{A^{\ell +1}}\gg {\left(\log\log t\right)}^{\ell +1}\;. \end{array}$$

Next, define

$$f\left(s\right):=\frac{\zeta^{\left(\ell \right)}\left(s\right)}{{\left(\log\log s\right)}^{\ell +1}}.$$

Suppose that $\zeta^{\left(\ell \right)}\left(1+it\right)\neq \Omega \left({\left(\log\log t\right)}^{\ell +1}\right)$. So f(1+it)=o(1). Clearly, f(2+it)=o(1). Then we get a contradiction with (44) by the Phragmén–Lindelöf principle (for instance, see[23, p. 189]).□

## 8 DISCUSSIONS, OPEN PROBLEMS, AND CONJECTURES

Let ℓ∈(0,∞) and σ∈R, define the following normalized log‐type GCD sums as:

$$\Gamma_{\sigma }^{\left(\ell \right)}\left(N\right):=\underset{|M|=N}{\sup}\frac{1}{N}\sum_{m,n\in M}\frac{{\left(m,n\right)}^{\sigma }}{{\left[m,n\right]}^{\sigma }}\log^{\ell }\left(\frac{m}{\left(m,n\right)}\right)\log^{\ell }\left(\frac{n}{\left(m,n\right)}\right)\;.$$

Problem 1 Given σ and ℓ, optimize $\;\Gamma_{\sigma }^{\left(\ell \right)}\left(N\right)$.

Remark 5 We are particularly interested in the case σ=1. Given ℓ, what is the optimal constant $C_{\ell }$ such that $\;\Gamma_{1}^{\left(\ell \right)}\left(N\right)⩽C_{\ell }\;{\left(\log_{2}N\right)}^{2\ell +2}$ ? *(*See [25] for both unconditional and conditional upper bounds*)*. When $\sigma \in (0,\frac{1}{2})$, is it true that $N^{1-2\sigma }{\left(\log N\right)}^{2\ell +\alpha (\sigma )}\ll \Gamma_{\sigma }^{\left(\ell \right)}\left(N\right)$ for some positive constant α(σ)? *(*These bounds are inspired by the work of Bondarenko–Hilberdink–Seip in [5], where the authors studied GCD sums for $\sigma \in (0,\frac{1}{2})$ *)*. It is not difficult to obtain the upper bounds that $\Gamma_{\sigma }^{\left(\ell \right)}\left(N\right)\ll N^{1-2\sigma }{\left(\log N\right)}^{2\ell +\beta (\sigma )}$ for some positive constant β(σ), by [5, Theorem 1] and arguments in the proof of Proposition *4* of [25].

We are also interested in extreme values of $|\zeta^{\left(\ell \right)}\left(\sigma \;+\;it\right)|$ in the left half strip. It is unlike the situation of the zeta function, where the values on the left half strip can be easily determined by the right half strip via the functional equation. Thus it is worth to study $\Gamma_{\sigma }^{\left(\ell \right)}\left(N\right)$ when $\sigma <\frac{1}{2}$, even for this reason.

Problem 2 Study extreme values of $\;|\zeta^{\left(\ell \right)}\left(\sigma \;+\;it\right)|$, when $\sigma \in (-\infty ,\;\frac{1}{2})$ and ℓ∈N are given.

We can use Theorem **A** of Ingham [16] to prove the following claim, from which we obtain the lower bounds (45) on maximum of $\;|\zeta^{\left(\ell \right)}\left(\sigma \;+\;it\right)|$. But we expect something slightly better.

Claim 1 Let ℓ∈N and $\sigma \in (-\infty ,\;\frac{1}{2})$ be fixed. Then

$$\int_{0}^{T}|\zeta^{\left(\ell \right)}\left(\sigma \;+\;it\right){|}^{2}dt∼{\left(2\pi \right)}^{2\sigma -1}\frac{\zeta (2-2\sigma )}{2-2\sigma }T^{2-2\sigma }{\left(\log\frac{T}{2\pi }\right)}^{2\ell },\;\;as\;\;T\to \infty .$$

In Theorem **A** of Ingham [16], let μ=ν=ℓ and a=b=σ, then

$$\int_{0}^{T}|\zeta^{\left(\ell \right)}\left(\sigma \;+\;it\right){|}^{2}dt=2\pi F_{2\ell }\left(\frac{T}{2\pi },2\sigma \right)+R_{\ell }\left(T,\sigma \right),$$

where

$$R_{\ell }\left(T,\sigma \right)=O\left(T^{\max\{1-\sigma ,\;1-2\sigma \}}{\left(\log T\right)}^{2\ell +2}\right)=o\left(T^{2-2\sigma }\right)$$

and

$$\begin{array}{cc} F_{2\ell }\left(T,2\sigma \right) & =\int_{1}^{T}\frac{\partial^{2\ell }}{\partial s^{2\ell }}\left(\zeta \left(s\right)+x^{1-s}\zeta \left(2-s\right)\right){|}_{s=2\sigma }dx\\ & ∼\zeta \left(2-2\sigma \right)\int_{1}^{T}x^{1-2\sigma }{\left(\log x\right)}^{2\ell }dx\\ & ∼\frac{\zeta (2-2\sigma )}{2-2\sigma }T^{2-2\sigma }{\left(\log T\right)}^{2\ell }. \end{array}$$

□

Immediately, we obtain the following corollary.

Corollary 1 Let ℓ∈N, β∈[0,1) and $\sigma \in (-\infty ,\;\frac{1}{2})$ be fixed. Then for large *T*,

(45) $$\max_{T^{\beta }⩽t⩽T}\left|\zeta^{\left(\ell \right)}\left(\sigma +it\right)\right|⩾\left(1+o\left(1\right)\right){\left(2\pi \right)}^{\sigma -\frac{1}{2}}\sqrt{\frac{\zeta (2-2\sigma )}{2-2\sigma }}T^{\frac{1}{2}-\sigma }{\left(\log T\right)}^{\ell }\;.$$

Note that the lower bound in Theorem 1 increases when ℓ increases. So it is natural to have the following conjecture.

Conjecture 1 If *T* is sufficiently large, then uniformly for all positive integers $\ell_{1},\ell_{2}$ ⩽ (logT) $\cdot {\left(\log_{2}T\right)}^{-1}$, such that $\ell_{1}<\ell_{2}$, we have

$$\begin{array}{c} \max_{T⩽t⩽2T}\left|\zeta^{\left(\ell_{1}\right)}\left(1+it\right)\right|<\max_{T⩽t⩽2T}\left|\zeta^{\left(\ell_{2}\right)}\left(1+it\right)\right|\;. \end{array}$$

When ℓ is fixed, we have the following conjecture, inspired by the conjecture of Granville–Soundararajan .

Conjecture 2 Let ℓ∈N be given. Then there exists a polynomial $P_{\ell +1}\left(x,y\right)$ of total degree ℓ+1 such that

$$\max_{T⩽t⩽2T}\left|\zeta^{\left(\ell \right)}\left(1+it\right)\right|=P_{\ell +1}\left(\log_{2}T,\;\log_{3}T\right)+o\left(1\right),\;\;as\;\;T\to \infty .$$

In particular, there exists a positive constant $c_{\ell }$ such that

$$\max_{T⩽t⩽2T}\left|\zeta^{\left(\ell \right)}\left(1+it\right)\right|∼c_{\ell }\cdot {\left(\log_{2}T\right)}^{\ell +1},\;as\;\;T\to \infty .$$

Remark 6 Does $\;\lim_{\ell \to \infty }c_{\ell }$ exist? In particular, do we have $\lim_{\ell \to \infty }c_{\ell }=0$?

Remark 7 When assuming the RH, one can get $\;|\zeta^{\left(\ell \right)}\left(1+it\right)|\ll_{\ell }{\left(\log_{2}t\right)}^{\ell +1}\;$ for sufficiently large t∈R (see [25]).

When ℓ∈N and σ∈(0,1) are given, we think that the maximum of derivatives of zeta function and maximum of zeta function only differs by multiplying some small factors. More precisely, we have the conjecture.

Conjecture 3 Let ℓ∈N and σ∈(0,1) be fixed, then there exists constants C(σ,ℓ) and c(σ,ℓ) which depend on σ and ℓ, such that for sufficiently large *T*, we have

$${\left(\log T\right)}^{c(\sigma ,\ell )}\cdot \max_{T⩽t⩽2T}\left|\zeta (\sigma +it)\right|\ll \max_{T⩽t⩽2T}\left|\zeta^{\left(\ell \right)}\left(\sigma +it\right)\right|\ll {\left(\log T\right)}^{C(\sigma ,\ell )}\cdot \max_{T⩽t⩽2T}\left|\zeta (\sigma +it)\right|,$$

where the implied constants depend at most on σ and ℓ. Moreover, when $\sigma \in (0,\frac{1}{2}]$, then we can take C(σ,ℓ)=ℓ+α(σ) and c(σ,ℓ)=ℓ+β(σ), where α(σ) and β(σ) are constants depending at most on σ.

When we try to give a different proof of Theorem 1 via Levinson's approach [18], we meet with the following problem. In particular, if the following problem has a positive solution, then a new proof for our Theorem 1 can be given.

Problem 3 Let ℓ∈N be given. Find n=n(k) and some positive constant $c_{\ell }⩾e^{\gamma }\cdot \ell^{\ell }\cdot {\left(\ell +1\right)}^{-(\ell +1)}$ such that if *k* is sufficiently large, then we have

$${\left(\frac{d_{k,\ell }\left(n\right)}{n}\right)}^{\frac{1}{k}}⩾c_{\ell }\cdot {\left(\log k\right)}^{\ell +1}+O\left({\left(\log k\right)}^{\ell }\right)$$

and

logn=klogk+O(k),

where $d_{k,\ell }\left(n\right)$ is defined as

$$d_{k,\ell }\left(n\right):=\sum_{m_{1}m_{2}\cdots m_{k}=n}{\left(\log m_{1}\right)}^{\ell }{\left(\log m_{2}\right)}^{\ell }\cdots {\left(\log m_{k}\right)}^{\ell }\;.$$

Remark 8 The arithmetic function $d_{k,\ell }\left(n\right)$ is not multiplicative, which makes the problem difficult.

Problem 4 Study extreme values of derivatives of L‐functions.

Problem 5 In our Theorem 1, we require $\ell ⩽\left(\log T\right){\left(\log_{2}T\right)}^{-1}$. What is the largest possible range for ℓ, that the result of Theorem 1 can still be valid. For instance, what can we say about the extreme values if ℓ=[T], or $\ell =[2^{T}]$?

Problem 6 Can one find some range for ℓ, such that the results in Theorem 2 can still hold?

Remark 9 The main terms always satisfy since we have $a_{\ell }\left(n\right)⩾1$ for all *n* and ℓ. It is not clear about the moments of derivatives of the zeta function if ℓ can depend on *T*. For instance, if we let $\ell =[\left(\log T\right){\left(\log_{2}T\right)}^{-1}]$, then what can we say about the second moments as T→∞,

$$\int_{0}^{T}|\zeta^{\left(\ell \right)}\left(\frac{1}{2}\;+\;it\right){|}^{2}dt∼\;?$$

When ℓ depends on *T*, it also seems difficult to bound the contributions of $\;\Delta^{+}+\Delta^{-}$ .

Moreover, we have the following general problem, which asks how large or how small the extreme values of $\;|\zeta^{\left(\ell \right)}\left(\sigma \;+\;it\right)|$ can be if ℓ can be taken arbitrary large with respect to the length *T* of the interval [T,2T].

Problem 7 Given $\sigma_{0}\in \left[0,1\right]$, decide which one of following four properties can be true.

(A) Given any function V:(0,+∞)→(0,+∞), there always exists some function $f_{V}:\;\left(0,+\infty \right)\to N$ such that if $\ell =f_{V}\left(T\right)$, then for sufficiently large *T*, we have

$$\max_{T⩽t⩽2T}\left|\zeta^{\left(\ell \right)}\left(\sigma_{0}+it\right)\right|\ll V\left(T\right)\;.$$

(B) Given any function V:(0,+∞)→(0,+∞), there always exists some function $f_{V}:\;\left(0,+\infty \right)\to N$ such that if $\ell =f_{V}\left(T\right)$, then for sufficiently large *T*, we have

$$\max_{T⩽t⩽2T}\left|\zeta^{\left(\ell \right)}\left(\sigma_{0}+it\right)\right|\gg V\left(T\right)\;.$$

(C) There exists some function V:(0,+∞)→(0,+∞), such that for all function f:(0,+∞)→N, if $\ell =f_{V}\left(T\right)$, then for sufficiently large *T*, we have

$$\max_{T⩽t⩽2T}\left|\zeta^{\left(\ell \right)}\left(\sigma_{0}+it\right)\right|\ll V\left(T\right)\;.$$

(D) There exists some function V:(0,+∞)→(0,+∞), such that for all function f:(0,+∞)→N, if $\ell =f_{V}\left(T\right)$, then for sufficiently large *T*, we have

$$\max_{T⩽t⩽2T}\left|\zeta^{\left(\ell \right)}\left(\sigma_{0}+it\right)\right|\gg V\left(T\right)\;.$$

## JOURNAL INFORMATION

*Mathematika* is owned by University College London and published by the London Mathematical Society. All surplus income from the publication of *Mathematika* is returned to mathematicians and mathematics research via the Society's research grants, conference grants, prizes, initiatives for early career researchers and the promotion of mathematics.
